# High-sensitivity computational miniaturized terahertz spectrometer using a plasmonic filter array and a modified multilayer residual CNN

**DOI:** 10.1515/nanoph-2023-0581

**Published:** 2023-11-02

**Authors:** Mengjuan Liu, Meichen Yang, Jiaqi Zhu, He Zhu, Yao Wang, Ziyang Ren, Yihui Zhai, Haiming Zhu, Yufeng Shan, Hongxing Qi, Junli Duan, Huizhen Wu, Ning Dai

**Affiliations:** Zhejiang Province Key Laboratory of Quantum Technology and Devices, School of Physics, and State Key Laboratory of Silicon and Advanced Semiconductor Materials, Zhejiang University, Hangzhou 310058, China; Hangzhou Institute for Advanced Study, University of Chinese Academy of Sciences, Hangzhou, Zhejiang 310024, China; University of Chinese Academy of Sciences, Beijing 100049, China; State Key Laboratory of Infrared Physics, Shanghai Institute of Technical Physics, Chinese Academy of Sciences, Shanghai 200083, China; Zhejiang Laboratory, Hangzhou 311100, China; Jiangsu Collaborative Innovation Center of Photovoltaic Science and Engineering, Changzhou 213164, China

**Keywords:** computational miniaturized terahertz spectrometer, plasmonic filters, adaptive deep-learning algorithm

## Abstract

Spectrometer miniaturization is desired for handheld and portable applications, yet nearly no miniaturized spectrometer is reported operating within terahertz (THz) waveband. Computational strategy, which can acquire incident spectral information through encoding and decoding it using optical devices and reconstruction algorithms, respectively, is widely employed in spectrometer miniaturization as artificial intelligence emerges. We demonstrate a computational miniaturized THz spectrometer, where a plasmonic filter array tailors the spectral response of a blocked-impurity-band detector. Besides, an adaptive deep-learning algorithm is proposed for spectral reconstructions with curbing the negative impact from the optical property of the filter array. Our spectrometer achieves modest spectral resolution (2.3 cm^−1^) compared with visible and infrared miniaturized spectrometers, outstanding sensitivity (e.g., signal-to-noise ratio, 6.4E6: 1) superior to common benchtop THz spectrometers. The combination of THz optical devices and reconstruction algorithms provides a route toward THz spectrometer miniaturization, and further extends the applicable sphere of the THz spectroscopy technique.

## 1 Introduction

Spectrometers are essential test instruments in various applications of biology, material identification and characterization, chemical analysis, and spectral imaging, etc. [1, 2]. Conventional benchtop spectrometers commonly depend on bulky dispersive optical components, long optical path lengths, and sophisticated interferometers to achieve high performance, thereby limiting their applications in handheld, portable, implantable, and on-chip scenarios [3, 4]. As an emerging strategy for spectrometer miniaturization due to the rapid development of artificial intelligence, computational spectrometers acquire unknown spectral information hidden in incident light by encoding it into a set of detectors using pre-calibrated response functions, instead of directly separating spectral components or implementing Fourier transform as conventional spectrometers do [5, 6]. There are two typical approaches to encode spectral information in computational spectrometers [7]. One approach is composite spectrum-to-space mapping, which distinguishes the spectral points (i.e., wavelength) through creating a signature one-dimensional (1D) or two-dimensional (2D) pattern in space domain for each wavelength using dispersive elements [8–10]. The other is spectral response engineering, which is to tailor a distinct spectral response for each detector. After the encoding, mathematical algorithms are demanded for decoding, i.e., spectral reconstructions [11, 12].

Over the past decade, miniaturized spectrometers have made great achievements in visible, near-infrared (NIR), mid-infrared (MIR), and long-wave infrared (LWIR) wavebands [13–17], however, there is a lack of reports in terahertz (THz) waveband, which is indispensable for security check, food quality inspection, biomedicine, etc. [18–20]. Since dispersive benchtop spectrometers (such as grating-based and prism-based spectrometers) are with badly low performance in THz waveband, the composite spectrum-to-space mapping is unbefitting for THz spectrometer miniaturization. Therefore, the other approach is the only option for developing computational miniaturized THz spectrometers, which has been maturely utilized for developing filter-array and filter-free spectrometers [14, 16]. The THz photon energy is such low that it is difficult to engineer spectral response of the detectors themselves, while optical elements are necessary. Among all categories of THz detectors, blocked-impurity-band (BIB) detectors are a type of promising competitor to be applied in THz spectrometer miniaturization because of their broad spectral response range, low noise, and high sensitivity, which are crucial for spectrometer performance [21–23].

Herein, we report a computational miniaturized THz spectrometer based on spectral response engineering, where hardware (light source, filter array, and detector components) and software (reconstruction algorithm) are involved. A high-performance phosphorus-doped germanium (Ge:P) BIB detector is employed to display spectral codes. Relying on theoretical simulations, we fabricate an optical element, i.e., a plasmonic filter array, to tailor the spectral response of the detector. Moreover, we propose a modified deep-learning algorithm for spectral reconstructions to adapt the optical properties of the filter array. The spectrometer exhibits workable spectral resolution and outstanding sensitivity. Our miniaturized THz spectrometer stimulates the development of spectrometer miniaturization in THz waveband that has significant application value in various fields.

## 2 Theoretical simulations

**Figure 1:**
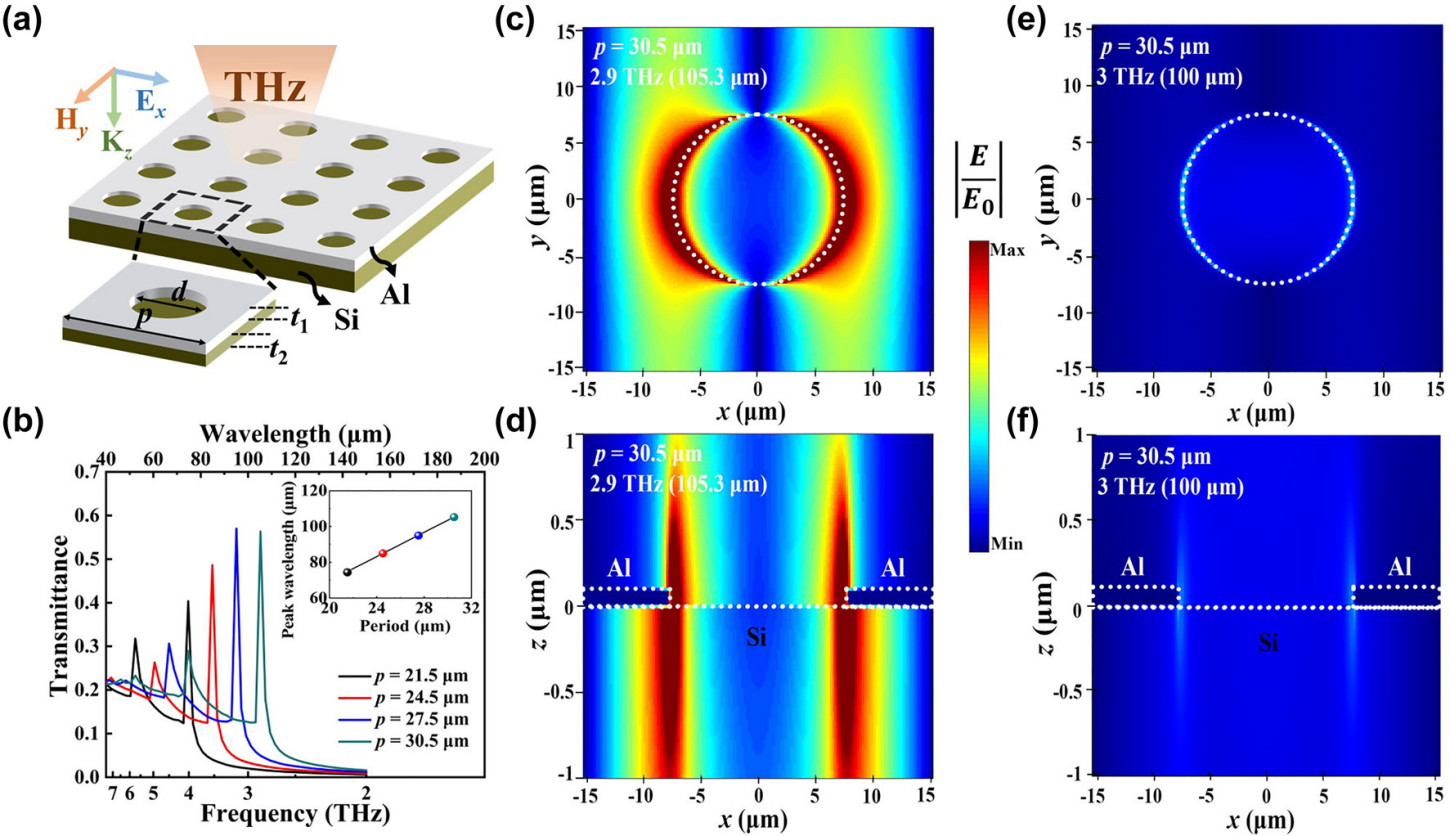
Theoretical simulations for the plasmonic THz filters. (a) Schematic diagram of a filter structure including a periodic square lattice array of Al holes and an ultrapure Si substrate. (b) Simulated transmission spectra of the filters with different periods in the array. Inset: the resonant wavelength in the transmission spectra as a function of the array period. (c) The electric field intensity mapping under the irradiation of monochromatic THz wave with the primary-peak frequency at the interface of the Al layer and Si substrate. (d) The electric field intensity mapping at the vertical cross-section. (e & f) the electric field intensity mapping corresponds to that in (c) and (d), respectively, at the non-resonant frequency. The color-scale bar represents the ratio of the enhancement electric-field and the incident electric-field magnitudes in (c–f).

Before experimental fabrications, we design the plasmonic THz filter structure and theoretically simulate the optical properties. Figure 1(a) schematically shows the designed filter structure, where an Al-based periodic hole array with a thickness *t*_1_ = 100 nm and an ultrapure silicon (Si) substrate with a thickness *t*_2_ = 300 μm are implemented. In the square lattice array, the hole diameter *d* is fixed as a half of the period *p*, and both of which are in subwavelength scale. Through using a commercial software, Finite-Difference Time-Domain (FDTD) Solutions, we simulate the transmission spectra and the electric-field intensity distributions of the filters with different periods. In the simulations, the optical property of metallic Al thin film is expressed by the Drude model with a plasma frequency *ω*_p_ = 3435.6 THz and collision frequency *γ*_0_ = 8.0 THz [24, 25], and the Si substrate is with a refractive index *n* = 3.42. To simulate the periodic arrangement with plenty of units more efficiently, periodic boundary conditions are set up along the *x* and *y* axes as displayed in Figure 1(a), and perfect-matching-layer (PML) boundary condition is set up along the *z* axis. The THz source is located at 80 μm above the Al layer surface, and *x*-polarized THz wave with a frequency range of 7.5–2.0 THz is normally incident onto the structure. The simulations are implemented on a discrete cubic mesh with a spacing of 0.2 μm in *x* and *y* axes, and 0.02 μm in *z* axis. Besides, the stopping criterion is set as 1.0E-5. A convergence analysis via varying the mesh is made, which indicates that reducing the mesh size is not able to obviously improve the simulation accuracy but increase the run time.

The simulated transmission spectra of the designed filters with different periods are shown in Figure 1(b). These spectra achieve the maximum transmittances at 4.0 THz (74.7 μm), 3.5 THz (84.9 μm), 3.2 THz (95.1 μm), and 2.9 THz (105.3 μm), respectively, which are the primary-peak frequencies of surface plasmon resonances (SPRs). According to the SPR effect, the THz wave is coupled with the surface plasmons formed at the Al/Si interface during the irradiation of the THz wave with a primary-peak frequency onto the structure. The transmittances at the primary-peak frequencies exceed unity when normalized to the area of the holes, and this phenomenon is the so-called extraordinary optical transmission [26, 27]. High-order resonance peaks are also observed in all the spectra, with larger full width at half maximums (FWHMs) and higher peak frequencies than the primary peaks. It is worth noting that the occurrences of the high-order peaks would weaken the filtering capability of the designed filters, and thus restrain the performance of the proposed computational miniaturized spectrometer. The curve of the primary-peak wavelength versus the array period is plotted in the inset of Figure 1(b), and a linear relation is presented with a slope ∼0.3 therein. This contributes to the design of the configurable filter array of our spectrometer. For a square lattice array, the SPR effect excited by normally incident irradiation theoretically follows the formula below [28, 29]:

(1) $$\lambda_{\text{p}}=\frac{p}{\sqrt{i^{2}+j^{2}}}\sqrt{\frac{\varepsilon_{\text{m}}\varepsilon_{\text{d}}}{\varepsilon_{\text{m}}+\varepsilon_{\text{d}}}}$$

where *λ*_p_ is the resonance peak wavelength, indices *i*, *j* = 0, 1, 2, …, are the scattering orders from the array, and *ε*_m_ and *ε*_d_ are the upper metal and the lower dielectric permittivities, respectively. The higher the SPR orders are, the lower the transmittance becomes.

To study the spatial evolution of the incident THz-wave energy when going through the plasmonic filter in the extraordinary optical transmission, we simulate the mapping of the electric field intensity around the designed structure in three dimensions (3Ds). Figure 1(c) shows the obtained mapping at the Al/Si interface in a period scale in the *xy* plane when the structure is irradiated by monochromatic THz wave with the primary-peak frequency. With the help of the SPR effect, the electric field intensity distributes mainly across the sidewalls of holes in *x* axis, exhibiting that the incident THz-wave energy is concentrated onto subwavelength scale there. In addition, the cross-section mapping in the *xz* plane is shown in Figure 1(d), where the longitudinal distribution of the field intensity is along the hole sidewalls. When the sample is irradiated by monochromatic THz wave, the incident THz wave could be absorbed by the Al layer and excite SPR wave. A portion of SPR wave becomes evanescent wave which vanishes away in the form of thermal loss, and the other is radiated from the Al/Si interface along the *z* axis. It is found from the longitudinal distribution that most of the radiation part of the incident THz energy is transmitted downward along the hole sidewalls while a fraction is reflected upward. There is almost no absorption of THz-wave in the ultrapure Si substrate, and thus the extraordinary optical transmission could occur in the above way. When the incident monochromatic THz wave is with a non-resonance frequency, the 3D mapping becomes as the shown in Figure 1(e) and (f). The vanishing of the SPR effect nearly eliminates the energy concentration and makes the diffraction limitation prominent. This leads to a distinct decline of the transmittance, and thus the extraordinary optical transmission disappears.

## 3 Fabrications and experimental measurement

**Figure 2:**
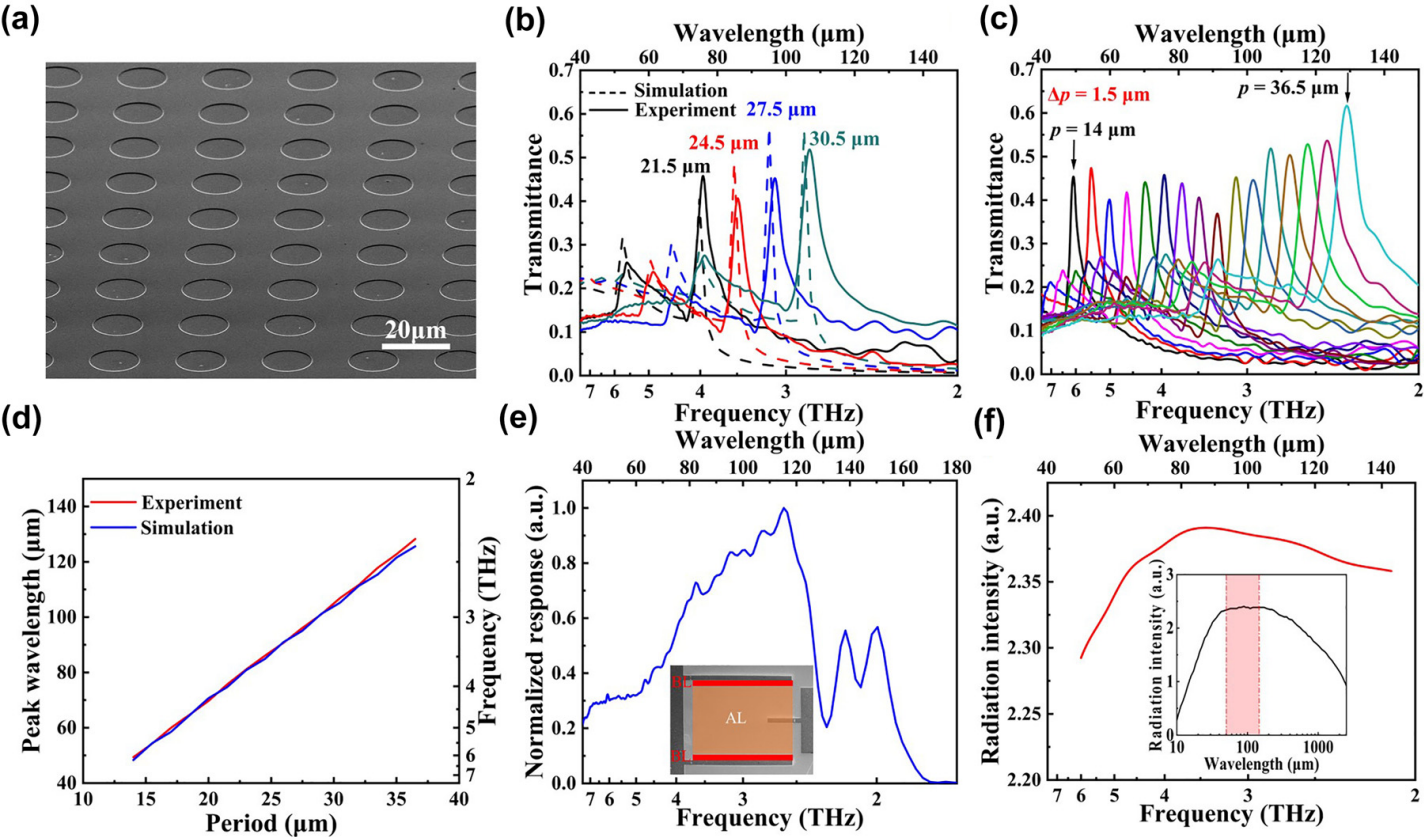
Optical properties of the fabricated hardware. (a) A skew-view SEM image of the periodic structure in a fabricated Al hole filter. (b) Comparison of experimental and simulated transmission spectra of the filters with different periods. (c) Measured transmission spectra of Al hole filters in the first row of the 16 × 15 filter array, with periods increasing from 14 μm to 36.5 μm with a step of 1.5 μm. (d) The measured and the simulated primary-peak wavelength as a function of the period. (e) Response spectrum of the Ge:P BIB detector. Inset: an SEM image of the detector with false-color AL and BL. (f) Source radiation spectrum within the working range of the spectrometer. Insert: the whole radiation spectrum of the Hg-arc light source, where the red shadow corresponds to the working range.

After designing the plasmonic THz filter structure, we fabricated a 16 × 15 plasmonic filter array with a whole array size of 4.96 × 4.65 cm^2^ on a 3-inch Si wafer, in which the periods of the elements increase from 14 μm to 37.9 μm with a step of 0.1 μm. The size of pixel of each filter is 0.31 cm; the period number of the minimum-period filter (*p* = 14 μm) is 217, while that of the maximum-period one (*p* = 37.9 μm) is 81. A 100 nm-thick Al layer is deposited on an ultrapure Si substrate wafer with a thickness of ∼300 μm using magnetron sputtering technique. Next, the sample surface is patterned with the filter array arrangement through electron-beam lithography, followed by a wet etching process carried out with Al corrosive liquid to configure the filter array onto the Si substrate. Thus, the fabrication of the filter array is completed. Figure 2(a) shows the skew-view scanning electron microscope (SEM) images of the periodic structure in a fabricated Al hole filter. Subsequently, the transmission spectra of the filter array are all measured using a commercial Fourier transform infrared (FTIR) spectrometer (Bruker VERTEX 80v) with a vacuum chamber, and then response to background radiation is corrected on these spectra. Corresponding to the simulated transmission spectra in Figure 1(b), the experimental spectra are given in Figure 2(b) for comparison. The resonance-peak frequencies of the experimental and the simulated spectra are in good consistence, while the experimental spectra exhibit obviously larger FWHMs than the simulated ones. The difference may arise from the variation of hole diameter and Al layer thickness, and defects in Al layer. On the other hand, although the limited period numbers of the filters can make the SPR effect excited, the filtering capability can be inevitably restrained compared with the simulation where limitless periods are involved, possibly leading to the above difference as well. The measured transmission spectra of Al hole filters in the first row of the 16 × 15 filter array are displayed in Figure 2(c). Moreover, the experimental and the simulated curves of the primary-peak wavelength versus the period over the whole filter array are plotted in Figure 2(d). They are highly consistent, presenting evident linear trends as theoretically forecasted referred to Eq. (1).

Besides the above fabricated filter array, a phosphorus-doped germanium (Ge:P) BIB detector is also developed and applied in the miniaturized spectrometer system. The fabrication procedure was presented in a previous work of us [30]. Since P is a shallow-level impurity element with ionization energy of 0.012 eV in Ge, the extrinsic photoelectric response of Ge:P falls in a THz waveband. Moreover, due to the existence of the blocked layer (BL) adjacent to the absorbing layer (AL) in the Ge:P BIB detector, the absorption coefficient of the AL can be effectively elevated by means of increasing the doping density while keeping low dark current [31]. In this case, the sensitivity of this detector is outstanding compared with other THz detectors, beneficial to the performance of the spectrometer. The photoelectric response spectrum of the Ge:P BIB detector is shown in Figure 2(e), where a waveband of 7.5–1.8 THz (40–165 μm) is covered, and thus all resonance wavelengths in the filter array lie in the response range. The detailed characterization methods for standard photoelectric properties (such as response spectrum, blackbody response, and dark current–voltage curve) of the detector were referred to the previous work [30]. In addition, light source is another essential component in this system as well. Considering applicable power distribution in the operating waveband of the spectrometer, a water-cooled Hg-arc source is selected as the THz illuminant in the spectrometer system. Figure 2(f) shows the source radiation spectrum within the operating range of the spectrometer, and the insert is the whole radiation spectrum of the Hg-arc light source, where the red shadow corresponds to the operating range.

## 4 Operational procedure

Different from other categories of spectrometers, our miniaturized spectrometer needs a primordial dataset including a number of known substance transmission spectra to train the reconstruction algorithm beforehand. We set up this dataset including 400 spectra with the same frequency range (6.0–2.1 THz) deriving from an open-source THz database, and these spectra are all measured using a commercial FTIR spectrometer [32]. The high spectral resolution of the FTIR spectrometer ensures that spectral features, such as peaks and shapes, are finely involved in the measured spectra, and hence it is suitable to regard these spectra as ground truths. The dataset is randomly and evenly divided into five sub-datasets, four of which are to train the algorithm, and the other one is for validation. In this case, a five-fold cross validation is allowed with successively using each of the five sub-datasets as the validation one, and thus the generalization of the spectral reconstructions can be evaluated. In order to improve the adaptability of the algorithm, the sizes of the five sub-datasets are augmented by using translation, rolling-over, and aliasing methods onto the spectra, such that more spectral characteristic information is contained in the new dataset. After the above augmentation, each sub-dataset eventually includes 1370 spectra.

**Figure 3:**
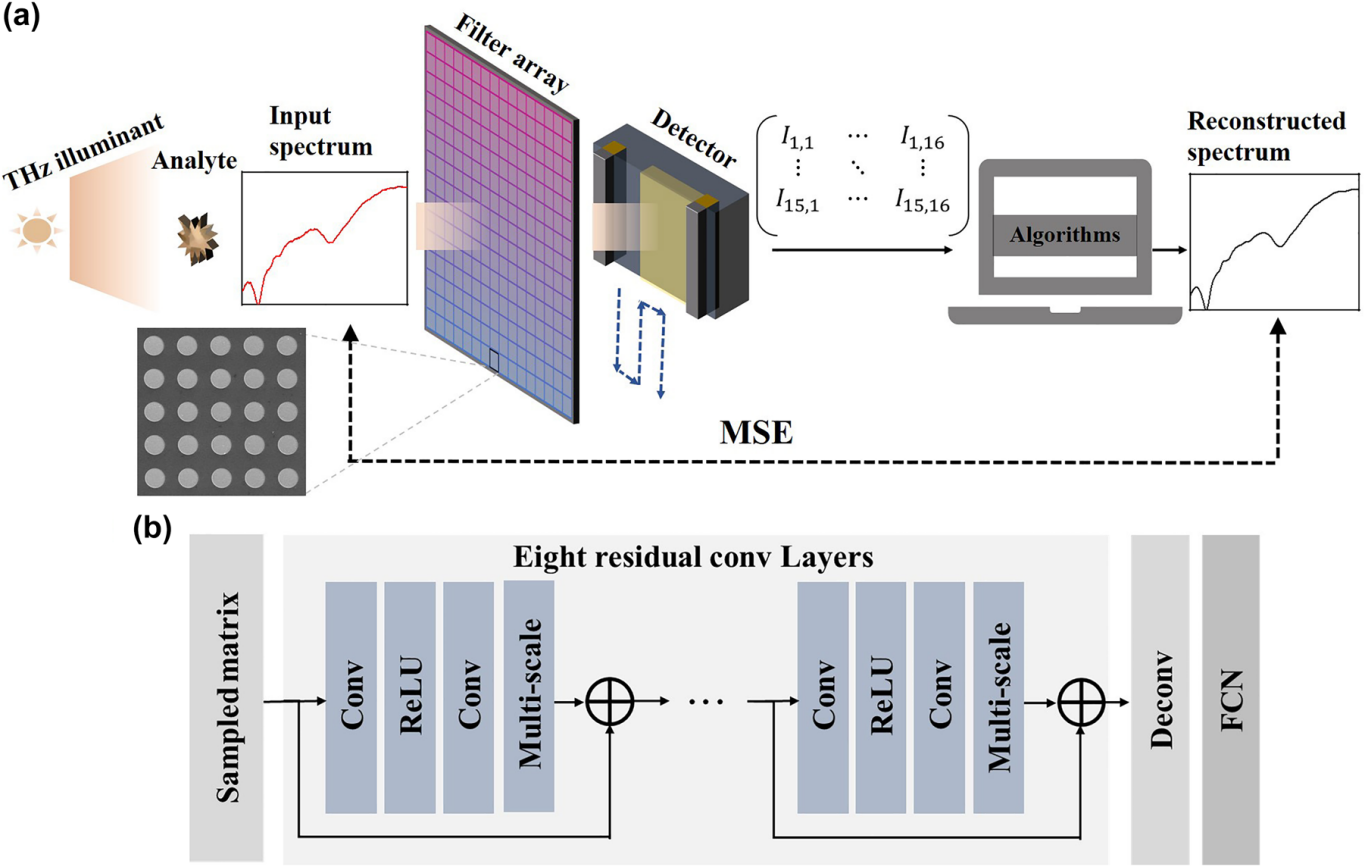
Operational procedure of our miniaturized spectrometer system. (a) The operational scheme of the computational spectrometer, involving the hardware (a THz illuminant, a filter array, and a detector) and the software (reconstruction algorithms). (b) The flowchart of the proposed AD-ResNet algorithm, where “Conv”, “ReLU”, and “Deconv” represent convolution, rectified linear unit, and deconvolution, respectively.

The operational procedure of the spectrometer system is displayed schematically in Figure 3(a). Firstly, the analyte is irradiated by the THz illuminant, and then the transmitted THz wave passes through the filter array. As stated above, the SPR occurs between the THz wave and the filters with different resonance peaks at this stage. That is, the power distribution of the THz wave radiated from the illuminant is modulated both in the space and the frequency domains by the filter array. Next, the modulated THz wave irradiates onto the Ge:P BIB detector mounted into a cryogenic dewar. After going through the photoelectric conversion process of the impurity atoms in the AL of the detector, the photocurrent signal can be collected from the detector, and this step is called as sampling. Since the THz wave is spatially modulated by the filter array, each filter corresponds to a photocurrent value of its own. With the aid of a motor-driving mechanism, the detector is precisely moved according to the filter elements behind all over the array to map the photocurrent matrix during the sampling, and thus the incident spectral information is encoded. This sampled photocurrent matrix can be mathematically written as:

(2) $$I_{kl}=\sum P\left(f\right)X\left(f\right)T_{kl}\left(f\right)R\left(f\right)$$

where *P*(*f*) is the radiation spectrum of the THz illuminant, *X*(*f*) is the transmission spectrum of the analyte, *T*_*kl*_(*f*) (indices *k*, *l* = 1, 2, …, 15/16, are the vertical and horizontal element numbers in the array) is the transmission spectrum of the detector-choose filter, and *R*(*f*) is the response one of the Ge:P BIB detector, respectively. What follows is inputting the sampled photocurrent matrix into the reconstruction algorithm, and finally the transmission spectrum of the analyte is reconstructed and outputted from the algorithm. When the miniaturized spectrometer is in practical applications, correction of background radiation is needed to be considered, and thus we propose a two-step sampling method to resolve this problem. Before putting the analyte in the system, a photocurrent matrix is sampled as the background signal; after analyte is introduced, second photocurrent matrix is sampled as the measuring signal. The difference between the two matrixes is then input into the algorithm for spectral reconstruction, and thus the background correction is executed in this way.

Note that before the matrix input, the reconstruction algorithm has been well trained. During the training and the validation, the sampled photocurrent matrix is obtained directly from Eq. (2) instead of collecting it from the Ge:P BIB detector. Thereinto, the mean square error (MSE) is employed as the loss function to evaluate the accuracy of the reconstructed spectra compared with the ground truths. The MSE loss is calculated according to the following equation:

(3) $$\text{MSE}=\frac{1}{N}\overset{N}{\underset{m=1}{\sum }}\left(\frac{1}{S}{\left\|{X^{\prime }}_{m}-X_{m}\right\|}_{2}^{2}\right)$$

where *N* is the spectrum total in the training sub-datasets, or the spectrum number in the validation sub-dataset, *S* is the point number in a spectrum, *X*_*m*_′ and *X*_*m*_ are the *m*th reconstructed and ground-truth spectra, respectively. Intense convergence displayed by the MSE loss with the increase of epochs indicates that the reconstruction algorithm is trained adequately. In the following spectral reconstructions for analytes with unknown transmission spectra, the MSE function is also utilized for evaluating the reconstruction quality, which is of the essence for the spectrometer performance.

As for the reconstruction algorithm, we employ a traditional algorithm, orthogonal matching pursuit (OMP) [33], and several deep-learning algorithms including super-resolution convolution neural network (SR-CNN) [34], enhanced deep super-resolution network (EDSR) [35], and auto-encoder deep neural network (AE-DNN) [36], respectively. It is worth mentioning that these deep-learning algorithms have been maturely applied in the 2D super-resolution imaging reconstruction field with satisfying performance. Moreover, considering that the transmission spectra of the fabricated THz filter array display relatively large FWHMs and obvious high-order resonance peaks, we propose an adaptive upsampling deep residual network (AD-ResNet) model. Figure 3(b) shows the flowchart of the AD-ResNet, which consists of eight residual convolutional layers for learning and extracting the spectral features from the input photocurrent matrix. To achieve the adaptive upsampling, we combine deconvolutional layers with linear fully connected network (FCN) layers. Note that the occurrences of the high-order peaks can generate spectral aliasing and distortion during the sampling process. If the characteristic peak of the measured analyte just locates at the high-order peaks of the filter array, the sampled photocurrent would be nonlinearly disturbed, making the correspondence between the sampled photocurrent and the reconstructed spectra more complicated. The AD-ResNet is with a fine ability to deal with the nonlinear relation, and it can automatically adapt and learn complex features of the input signal. For eliminating the detrimental impact on the spectral reconstruction, we abandon jump structures that can merge low-level feature information, not as the imaging reconstruction algorithms do, and besides, we also add a multi-scale layer in each residual convolution layer to keep the network stable. These alterations also make the network learn more information about the mapping relation between the output result and the deeply hidden features of the ground truth with lesser redundancy. Therefore, the AD-ResNet is supposed to be more suitable for THz spectral reconstructions.

The utilized algorithms are trained using the Adam optimizer with an initial learning rate of 1E-3. All preprocessings and trains/validations of the algorithms are performed with PyTorch libraries. All spectral reconstructions are performed on a server computer with two NVIDIA RTX3090 graphics processing card.

## 5 Performance presentation

**Figure 4:**
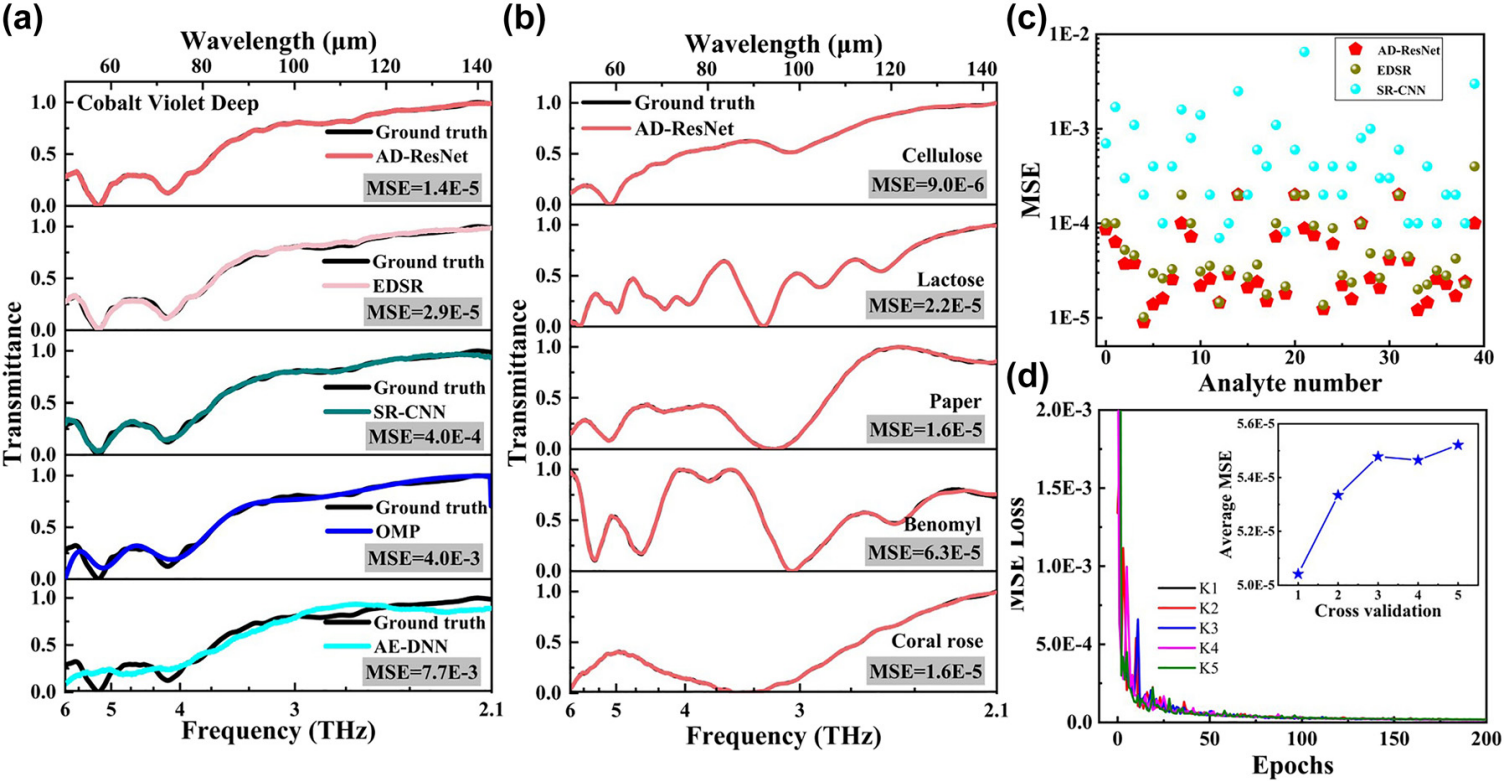
Spectral reconstruction performance of the spectrometer system. (a) The transmission spectra of the same cobalt violet deep analyte reconstructed by the spectrometer with the different algorithms and their ground truths. (b) The transmission spectra of five different analytes reconstructed by the spectrometer with the proposed AD-ResNet and their ground truths. In (a) and (b), the MSEs for evaluating the reconstruction quality are given. (c) MSE distributions for the 40 additional analytes obtained with the deep-learning algorithms. (d) The MSE loss versus epoch during the trainings with the AD-ResNet in the five-fold cross validation, where K1 represents the regular training, K2–K5 represent other folds; inset: the average MSE for the test dataset in different folds.

After the trainings of the reconstruction algorithms are completed, 40 additional analytes with unknown transmission spectra are measured using this spectrometer system to characterize its reconstruction capability with different algorithms. Figure 4(a) shows the reconstructed transmission spectra of the same analyte (i.e. cobalt violet deep) obtained using our spectrometer system with the different algorithms and the corresponding ground truth as well. Similar to the primordial dataset, the ground truths of the 40 analytes are also the measured transmission spectra with the FTIR spectrometer, and they are then used to build a new dataset named as test dataset. According to the meticulous curve comparisons of the reconstructed and the ground-truth spectra for the different algorithms displayed in Figure 4(a), the AD-ResNet reconstruction resolves the analyte transmission spectrum with the best accuracy among these algorithms, which is also visually manifested by its smallest MSE given over there. The AE-DNN reconstruction has the largest MSE, hence presenting the worst accuracy with a failure in revealing the two absorption peaks at 5.2 THz and 4.1 THz, respectively. We guess the reason is that the training in the AE-DNN reconstruction is badly impacted by the high-order peaks of the filter array. As for the other three algorithms, the fundamental spectral information (e.g., the two absorption peak positions and intensities) is basically restored in their reconstructions, yet the SR-CNN and the EDSR reconstructions display much higher precision than the OMP one. Note that the spectral reconstructions with traditional algorithms rely on multi-iterations to improve the precision instead of trainings, and thus the reason that the deep-learning algorithms (except the AE-DNN) behave better than the OMP is possibly due to the sufficient trainings implemented in the deep-learning reconstructions. Not only that, each reconstruction with traditional algorithms needs to take a quite long time to operate the multi-iterations, while the deep-learning reconstructions can output results much more quickly once the algorithms are well trained.

The transmission spectra of five different analytes randomly picked from these measured analytes reconstructed with the AD-ResNet and the corresponding ground truths in the test dataset are shown in Figure 4(b). It is clearly seen that the curves of the reconstructed spectra and the corresponding ground truths are fully consistent, indicating that the elaborate features of the ground truths are all presented in the reconstructed spectra. Therefore, the spectral reconstructions with the AD-ResNet possess a greatly high precision. Referred to the test dataset, the MSEs of these measured analytes using the deep-learning algorithms are calculated and given in Figure 4(c). These MSE distributions visually reveal that our AD-ResNet performs better than the other deep-learning algorithms throughout the whole test dataset instead of for some specific analytes. More precisely, the average MSEs of the AD-ResNet, the SR-CNN, and the EDSR are 5.0E-5, 9.1E-4, and 7.5E-5, respectively. On the whole, the MSEs of the AD-ResNet reconstructions are steadily below 2.0E-4, manifesting a promising practical potential of our spectrometer system. To evaluate the generalization of the AD-ResNet reconstructions, the five-fold cross validation is carried out. The MSE loss variations as epoch increases in all folds are shown in Figure 4(d), from which it can be learned that all the curves are basically coincident. Besides, the inset of Figure 4(d) shows the average MSE for the test dataset in different folds, where the average MSE keeps stable with a tiny variation amplitude of below 1.0E-5. Hence, the ground truths for the AD-ResNet training are not cherry-picked, and the spectral reconstructions are indeed generalizable. Incidentally, the MSE loss dramatically declines and then shows rapid convergence within 200 epochs, such that the trainings are sufficiently reliable. According to the timekeepings during the trainings for the deep-learning algorithms, the training times of the AD-ResNet, the SR-CNN, and the EDSR in 300 epochs are 8233 s, 9950 s, and 29,720 s, respectively, indicating that our proposed model is more easily trained than the other ones.

Subsequently, we study the spectral resolution of our spectrometer system. The spectrometer is far different from FTIR spectrometers and THz time-domain spectroscopy (THz-TDS) instruments in operational mechanism, which belong to conventional instruments for identifying analytes in THz waveband. Instead, the internal structure in our system equipped with the filter array for modulating incident THz wave is similar to dispersive spectrometers to a certain degree. Consequently, we refer to a standard spectral-resolution calibration method commonly used in dispersive spectrometers to characterize our spectrometer. Firstly, multiple monochromatic spectra with different FWHMs are simulated as the radiation spectra, and then the sampling is carried out based on Eq. (2). Next, the sampled photocurrent matrixes are input into the well-trained deep-learning algorithms for spectral reconstructions. The FWHM of the output spectrum is supposed to decrease gradually with that of the simulated spectrum, and then tends to be stable when that of the simulated spectrum is small enough. At this juncture, the FWHM of the output spectrum can be regarded as the spectral resolution, which is also called as the spectral bandpass.

**Figure 5:**
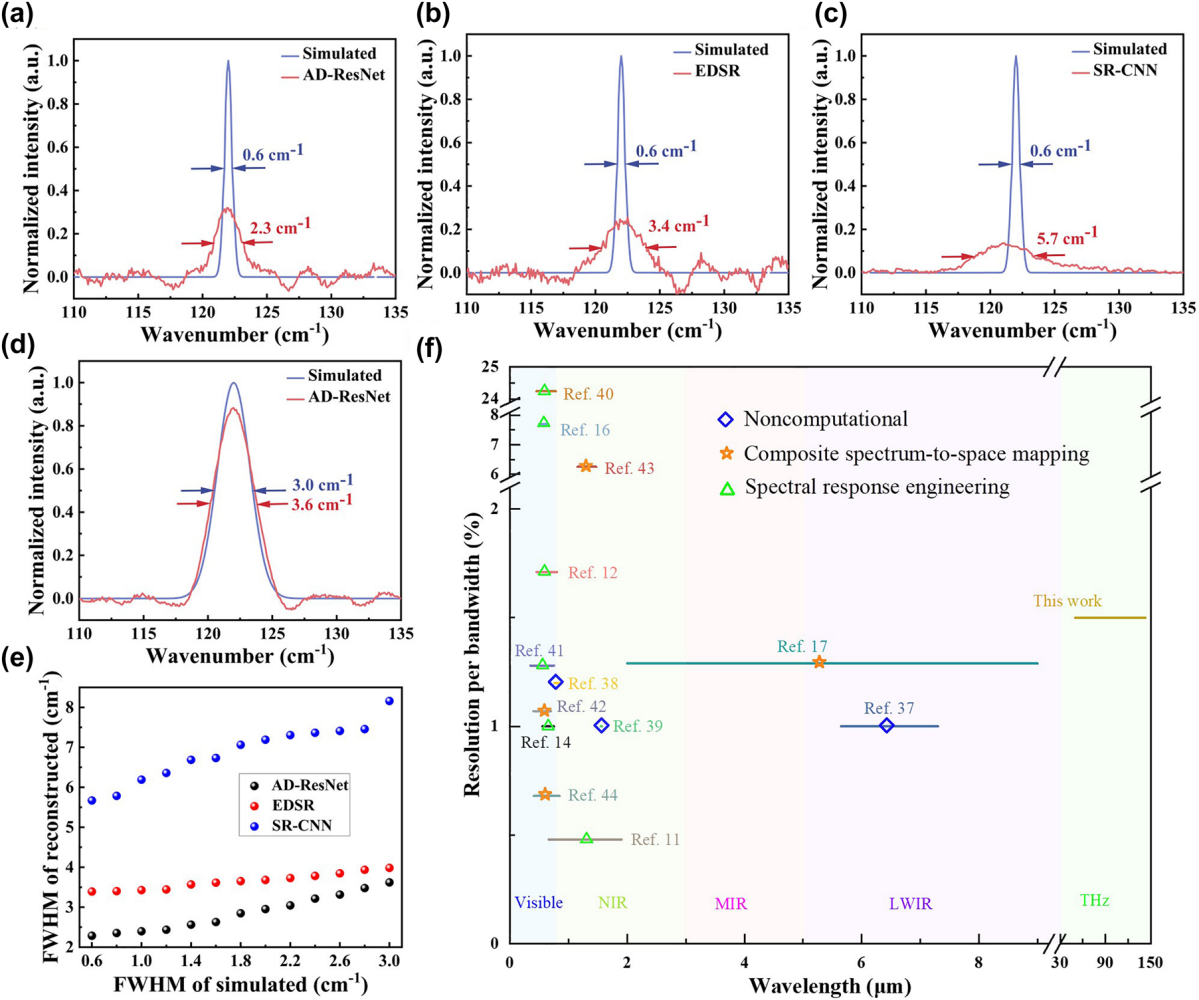
Spectral resolution presentation. (a–c) The reconstructed spectra using the deep-learning algorithms when the simulated spectra are with the same FWHM of 0.6 cm^−1^. (d) The reconstructed spectra using the AD-ResNet when the simulated FWHM is 3.0 cm^−1^. (e) The FWHMs of the output spectra versus those of the simulated spectra for the deep-learning algorithms. (f) The comparison of the spectral RRB of our spectrometer with other miniaturized spectrometers [11, 12, 14, 16, 17, 37–44].

When the FWHM of the simulated spectrum is 0.6 cm^−1^, the output spectra reconstructed using the deep-learning algorithms are displayed in Figure 5(a)–(c). The reconstructed spectra present different FWHMs for different algorithms, demonstrating that the spectral resolution is influenced by the reconstruction algorithms. In addition, the peak intensities of all the reconstructed spectra are weaker compared with the simulated ones, because the ultra-narrow simulated spectra make the sampled photocurrents with small magnitude. As the FWHM increases, the simulated and the reconstructed curves are gradually accordant, as shown in Figure 5(d). After multiple reconstructions for the simulated spectra, we acquire the relation between the FWHMs of the output and the simulated spectra for the deep-learning algorithms, which is displayed in Figure 5(e). From this presentation, it can be learned that the spectral resolutions for the AD-ResNet, the EDSR, and the SR-CNN are 2.3 cm^−1^, 3.4 cm^−1^, and 5.7 cm^−1^, respectively, again proving that our proposed AD-ResNet performs better than the other algorithms. In spectral resolution, although our spectrometer is inferior to conventional benchtop THz spectrometers, it rivals and even overmatches commercial THz spectrometers based on attenuated total reflection (ATR) including benchtop and handy probe types, which are produced by Hamamatsu Photonics [45]. The satisfying spectral resolution makes our spectrometer applicable to identify analytes in most practical scenarios.

For spectrometers with different operational wavebands, the ratio of spectral resolution to bandwidth (RRB) (both of spectral resolution and bandwidth are in units of wavelength) can be treated as a standard for performance comparison, where the bandwidth is represented by the spectral range. The lower the RRB, the better the performance on resolved capability. The RRB of our spectrometer is calculated as 1.5 %, and the RRB comparison for our spectrometer and other miniaturized spectrometers (working in visible, NIR, MIR, and LWIR wavebands) is presented in Figure 5(f). The RRB of our spectrometer is at a moderate level among these miniaturized spectrometers, yet note that its operational wavelengths are far longer than the others.

Besides, we also study the sensitivity of the spectrometer. The signal-to-noise ratio (SNR) mainly depends on the Ge:P BIB detector and the filter array in our system because of the inexistence of bulky optical components. According to the detector sensitivity reported in Ref. [30] and the transmission spectra shown in Figure 2(c), the SNR is calculated to be 6.4E6: 1. This ultrahigh level of the SNR is due to the ultralow noise equivalent power (NEP) of the detector (3.6E-15 W/Hz^1/2^), and it can even be further improved via enhancing the irradiance of the THz light source. Unfortunately, because of the non-negligible MSEs in the spectral reconstructions, the SNR is supposed to be reduced, yet the average MSE reaching 5.0E-5 makes sure that the reduction is within one order of magnitude. Even so, the reduced SNR is still superior to benchtop THz spectrometers, including several FTIR types with SNR 6.0E4: 1 [46], 6.5E4: 1 [47], 5.5E4: 1 [48], and TDS types with SNR 1.0E5: 1 [49], 1.8E4: 1 [50], 1.0E4: 1 [51]. The SNR superiority of our spectrometer arises from the Ge:P BIB detector compared with the FTIR type using pyroelectric detectors and the THz TDS type using photoconductive antenna detectors. As is known, the SNR decreases with the concentration of the analyte, and when the SNR decreases to 3:1, the corresponding analyte concentration is generally defined as the detection limit (DL) of spectrometers. Benefitting from the excellent SNR, the theoretical DL of our spectrometer can achieve 0.5 ppm. Moreover, dynamic range, the ratio of the maximum and the minimum of detectable signals for spectrometers, is studied as well. In our spectrometer, the detectable maximum signal corresponds to the peak response of the detector, and the detectable minimum signal is equal to the detector noise. Hence, the dynamic range is the same as the SNR at the peak frequency of 2.6 THz, i.e., 2.7E7: 1, which is also superior to the FTIR and the THz TDS spectrometers. The overall performance of the spectrometer, including spectral resolution and sensitivity, is listed in Table 1. The proposed spectrometer with outstanding performance carries forward the reconstructed miniaturization strategy from LWIR into THz wavebands. Although our spectrometer does not conform the requirement of implantable and on-chip integrated applications due to the separation of the optical element and the detector, it still has a significant meaning in handheld and portable applications.

**Table 1:** The overall performance of our miniaturized spectrometer.

Frequency range	Average MSE	Spectral resolution	RRB	SNR	DL	Dynamic range
6.0–2.1 THz	5.0E-5	2.3 cm^−1^	1.5 %	6.4E6: 1	0.5 ppm	2.7E7: 1

## 6 Conclusions

In summary, we have demonstrated a computational miniaturized THz spectrometer, where a light source, a plasmonic filter array, a Ge:P BIB detector, and an AD-ResNet reconstruction algorithm are involved. The filter array engineers the detector response for encoding the incident spectral information, and then it is decoded by the AD-ResNet. The transmission spectra of the filter array show large FWHMs and high-order resonance peaks, and this disadvantage onto the spectral reconstructions is suppressed by the AD-ResNet compared with several deep-learning algorithms full-fledged in the 2D super-resolution imaging field. The spectral resolution of the spectrometer achieves a satisfying level of 2.3 cm^−1^, and it even overmatches common benchtop THz spectrometers in sensitivity thanks to the utilization of the Ge:P BIB detector. The demonstration of our spectrometer blazes a trail for miniaturized THz spectrometer, and thus promotes the application of the THz spectroscopy technique in handheld and portable scenarios.
